# Salinisation of drinking water ponds and groundwater in coastal Bangladesh linked to tropical cyclones

**DOI:** 10.1038/s41598-024-54446-6

**Published:** 2024-03-03

**Authors:** ChiSan Tsai, Mohammad A. Hoque, Paolo Vineis, Kazi Matin Ahmed, Adrian P. Butler

**Affiliations:** 1 Department of Civil and Environmental Engineering, Imperial College London, London, UK; 2 Department of Environmental Systems Graduate School of Frontier Sciences, The University of Tokyo, Tokyo, Japan; 3 School of the Environment, Geography & Geosciences, University of Portsmouth, Portsmouth, PO1 3QL UK; 4 Department of Epidemiology and Biostatistics, Faculty of Medicine, MRC-HPA Centre for Environment and Health, Imperial College London, London, UK; 5 Department of Geology, University of Dhaka, Dhaka, Bangladesh

**Keywords:** Environmental sciences, Hydrology, Natural hazards

## Abstract

Salinity is a widespread problem along the Asian coast, mainly in reclaimed lands where most people live. These low-lying areas are vulnerable to impacts from tropical cyclone induced storm surges. The role of such surges on the long-term salinity of water resources, particularly the salinisation of drinking water ponds, a key water resource, requires further investigation. Here we show, using high-resolution measurements of pond hydrology and numerical modelling, that episodic inundation events cause the widespread salinisation of surface water and groundwater bodies in coastal areas. Sudden salt fluxes in ponds cause salinity build-up in the underlying sediments and become a source of salinity. Rapid clean-up of drinking ponds immediately after a surge event can significantly minimize these salinity impacts, which are likely to increase under climate change. Our study has implications for coastal land use and water resources management in tropical deltas.

## Introduction

**Figure 1:**
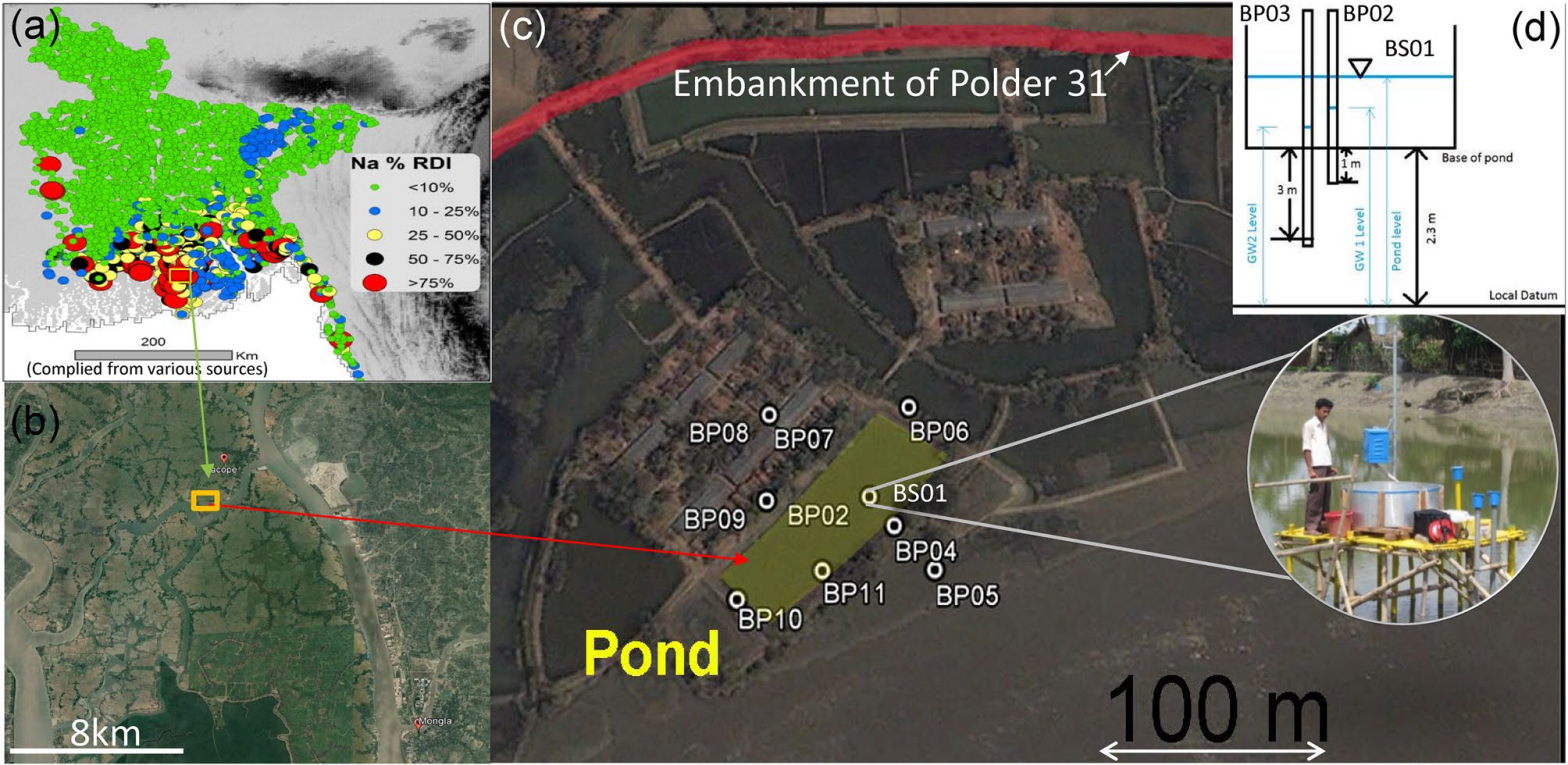
Location and monitoring of study area. (**a**) Sodium (Na) content in groundwater shown as percentage of recommended daily intake (RDI) (based on the consumption of 2 L per person per day). Modified from Fig. 4a, Hoque and Butler^32^ (background topographic elevations from EROS, 2002. Shuttle Radar Topography Mission (SRTM) Elevation Data Set). (**b**) Location of study area in Dacope Upazila. (**c**) Monitoring sites around DAB Pond, located just outside of embankment for Polder 31, with details of monitoring instrumentation within pond (**d**) (BS01 was set to represent the measurement taken at the pond, BP02 was set to represent the measurement obtained from the groundwater at a depth of 1 m below the pond base and BP03 was set to represent the measurement obtained from the groundwater at a depth of 3 m below the pond base). Images used in (b) and (c) sourced from Google Maps (2020) Bangladesh, available at: http://maps.google.co.uk (Accessed: 5 July 2020). Inset photograph in (c) by M. Hoque.

Salinity in water and soil, like many other deltaic coasts in the world^1,2^, is pervasive in the low-lying coastal area of Bangladesh, home to around 35 million people^3,4^. The soil salinity affects 20–30% of net cultivable land causing productivity decline^5,6^, and drinking water salinity^7–9^ has an association with prevalent hypertension^10–13^—a condition associated with pre-eclampsia^14–16^ in pregnant women and cardiovascular diseases in general, and increased rate of infant mortality^17^. Many people live in reclaimed land^18,19^ (locally known as polders). These comprise an area of approximately 25,000 km^2^, which is within a 2-m height of sea level. The polders are protected by up to 4-m high embankments to prevent flooding by saline tidal water. Even with this protection, the area is vulnerable to salinisation. Within polders, there is economically favourable but environmentally damaging shrimp cultivation^20,21^ where saline water is deliberately maintained in surface ponds. Externally, the low-lying topography, wide river estuaries and concave coastal geometry makes the region vulnerable to landfalling cyclones. Storm surges associated with these cyclones can result in overtopping or breaching of polder embankments leading to widespread inundation by seawater^22^. Bangladesh is impacted by cyclones circa every 3 years^23^ and the occurrence is increasing^24–26^ In recent times, Cyclones Sidr (Nov 2007), Aila (May 2009), Mahasen (May 2013), Komen (Jul 2015), Fani (Apr 2019), and Amphan (May 2020) (Table 1) made landfall causing widespread inundation due to embankment overtopping and failure. Furthermore, where embankment breaches have occurred, delays in repair result in affected areas being flooded twice a day by tidal water over long periods^19,27^, and causing widespread salinization of soil and drinking water storage ponds^28^. For example, breaches caused by Cyclone Aila resulted in many ponds in Polders 31 and 32 (located at Khulna district in Supplementary Fig. S.3) being inundated with saline water making them undrinkable and with major impacts on the health and wellbeing of those affected^10,14,15^. Our survey of ponds in Polders 31 and 32 (Fig. S.3), showed many having salinities between 1000 and 3000 mg/L, in excess of the WHO health limit. In some cases these high salinities were following an attempt to remediate (i.e. empty and refill) the pond. These long-term, high salinities in drinking water ponds are causing a social crisis in coastal regions^29,30^. Therefore, further investigation is required to test if the transport of saline water by cyclones is responsible for the elevated salinity levels in ponds and groundwater in coastal Bangladesh. The objectives of this paper are: (1) to gain insight into the interaction between surface water bodies (ponds) and groundwater salinities by conducting field measurements and numerical modelling in the Dacope Upazila of southwest coastal Bangladesh (Figs. 1b and S.1) and (2) to assess the effects of climate change induced by different frequencies of the tropical cyclones on the shallow groundwater salinities. ^25^In addition, we investigate, through numerical simulation, two pond remediation scenarios to demonstrate the effectiveness of post-storm surge pond remediation in reducing pond water salinity.

**Table 1:** Major tropical cyclones hit Bangladesh recently^31^.

Cyclone	Category*(wind speed)	Date
Cyclone Sidr	Category 5 (251 km/h or higher)	Nov 2007
Cyclone Aila	Category 1 (119–153 km/h)	May 2009
Cyclone Mahasen	− (85 km/h)	May 2013
Cyclone Komen	Category 1 (119–153 km/h)	July 2015
Cyclone Roanu	Category 1 (119–153 km/h)	May 2016
Cyclone Mora	Category 1 (119–153 km/h)	May 2017
Cyclone Fani	Category 4 (209–251 km/h)	May 2019
Cyclone Amphan	Category 4 (209–251 km/h)	May 2020

*Classified by the Saffir–Simpson hurricane scale based on the highest wind speed.

## Results

### Pond hydrology

**Figure 2:**
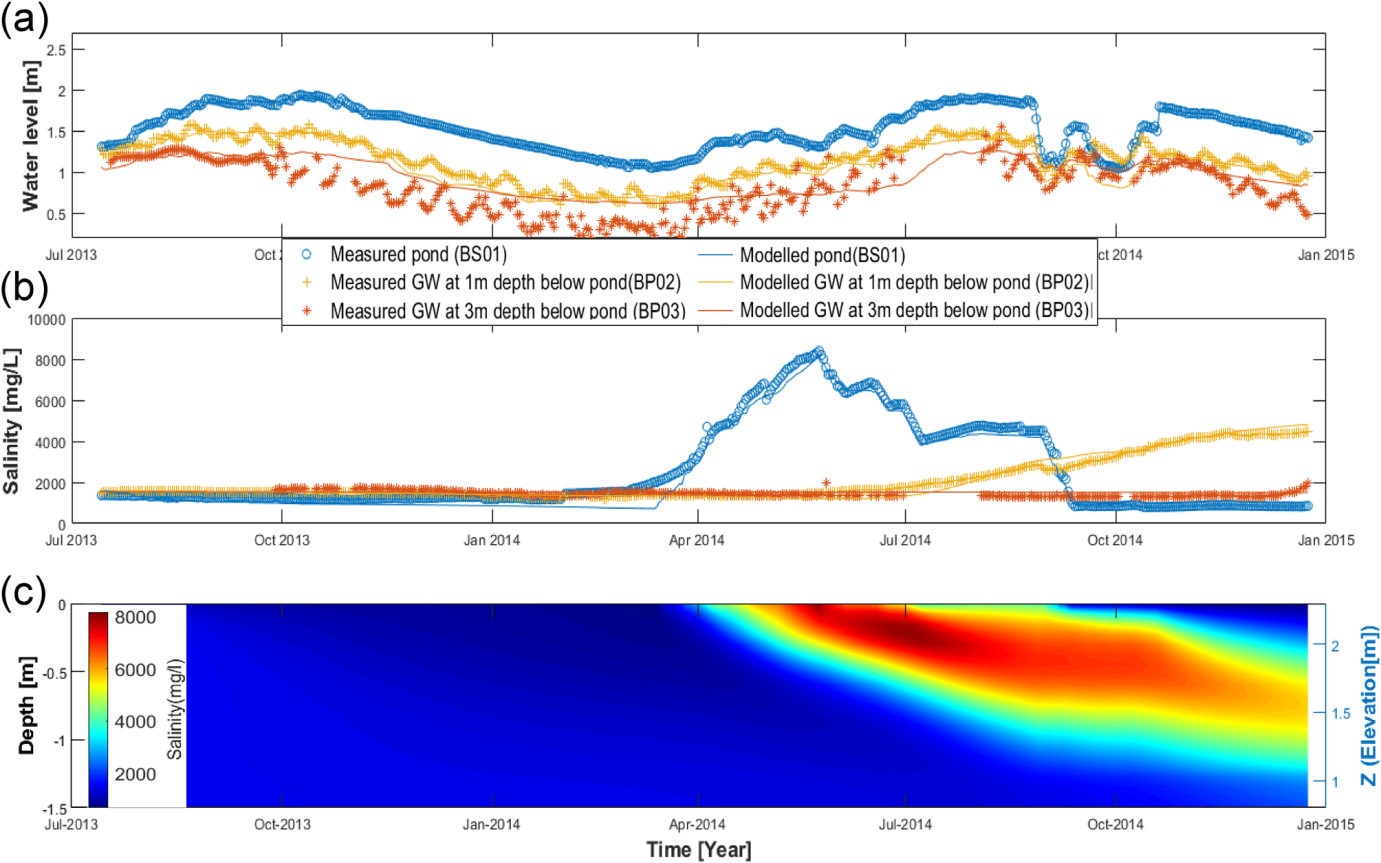
Simulated (symbols) and observed (lines) water levels (**a**) and salinity (mg/L) (**b**) in pond (blue), groundwater at 1 m (yellow) and at 3 m (orange) depths. (c) Simulated salinities down to 1.5 m below base of pond during monitoring period (July 2013 to Jan 2015).

An instrumented drinking water pond (labelled BS01 in Fig. 1c,d) in the Dacope Upazila of southwest coastal Bangladesh has been simulated using a numerical process-based model (see Methods section). Comparison of rainfall (SI Fig. S.2a) and pond water level data (BS01) (blue line in Fig. 2a) shows that rises in the pond water level are almost equal to the rainfall amount. This implies that there is no additional run-on to the pond from the surrounding area. Water loss from the pond occurs due evaporation or abstraction. A further loss is through seepage to underlying sediments. Water exchange through seepage can occur between the pond and the groundwater in the underlying sediments. Logged groundwater levels at 1m depth below the pond base (yellow line in Fig. 2a) show semi-diurnal and fortnightly fluctuations, due to hydraulic loading from the adjacent tidal river, combined with a season response that follows pond water levels^33^ (Supplementary Fig. S.2a). There is a general downward head gradient of 0.3 to 0.5 between the pond base and 3m depth (orange line in Fig. 2a), which implies a loss of water from the pond due to seepage. The sediments underlying the pond are predominantly clay to silty-clay with an estimated hydraulic conductivity of 2–3 mm/d^9^. This gives a daily seepage loss of around 0.6–1.5 mm, which is consistent with observations elsewhere in Bangladesh^9^. The seepage rate was also determined using a water balance calculation for the pond over the period 15 July 2013–14 July 2014 (Supplementary Table S.1) and gave a value of 1.7 mm/day, which compares well with above estimate^26^.

### Pond and groundwater salinity

The above fluxes have differing effects on pond salinity. Abstraction and seepage leave salinity unchanged, whereas rainfall causes a decline through dilution, and evaporation produces a rise through distillation. As the water depth is typically around 1–2 m (Fig. 2a), these changes in salinity are relatively small but observable, as can be seen during the period July 2013 to March 2014 (Fig. 2b). Here, a seasonal variation in salinity (ranging between 1000 and 1500 mg/L (i.e. blue line in Fig. 2b) was observed due to rainfall dilution^34^ (see Supplementary Fig. S.2A) during the monsoon July 2013 to Sep 2013 and re-concentration through evaporation loss over the dry season Oct 2013 to March 2014 (blue line in Fig. 2b).

Of particular concern, however, are episodic influxes of saline water due to storm surges and their long-term effect on pond salinities. Such occurrences have major impacts on drinking water quality and on the health and well-being of those affected^27^. Whilst no such event occurred during the period the pond was monitored, there was a sudden and sustained increase in salinity that occurred between 14 March 2014 and 24 May 2014 (blue line (BS01) in Fig. 2b). This was due to a failure in a pond levee that separated the drinking water pond from an adjacent shrimp farm pond^35^ with salinities around 15,000 mg/L. A major inflow of saline water occurred between 16 March and 24 May 2014 shown by a 0.3 m rise in pond water level during the dry season. This was accompanied by a sharp rise in pond salinity (BS01) from 2000 mg/L to a peak of over 8000 mg/L in June 2014. At this point the monsoon season started, causing dilutions during rainfall events, which were followed by rises in salinity during intervening dry periods. This sequence of falls and rises continued until September 2014, when steps were taken to remediate the pond. This was achieved, during the following two months, by flushing the pond with river water during high tide and the draining it during low tide. This process enabled pond salinities to be reduced to around 800 mg/L, so that it could be used for drinking water once again. Figure 2c shows groundwater salinities distributions from the pond base down to a depth of 1.5 m within the underlying sediment which implies the build-up of salinity in shallow groundwater due to the influx of saline water.

Confirmation of downward seepage of the pond water into the underlying alluvial sediments, as described above, was provided by the logged conductivities at 1 m depth. Between July 2013 and March 2014 groundwater salinities at 1m depth (BP02 yellow line in Fig. 2b) were similar to those in the pond. Following the intrusion of saline water into the pond, however, there was a rise in groundwater salinity at 1m depth, which occurred around 3–4 months after the initial rise in pond salinity and a peak arrival estimated at around 6 months. Using an approximate value of 5 months, gives a pore water velocity of around 6 mm/d and, assuming an effective porosity of 0.3^9^, a seepage rate of around 2 mm/day, which is comparable with the estimates derived from the water balance calculations.

### Modelling

The steady state model (Supplementary Fig. S.6b) provided the initial conditions for the dynamic simulation model. Rainfall, abstraction, and evaporation data provided the main hydrological drivers. As discussed above, there are a range of controls on pond salinity. In addition to a reduction by rainfall dilution and an increase through evaporation, there are more complex exchanges, via diffusion and advection, with the groundwater^36^ directly beneath the pond. The interaction between pond water and groundwater is largely controlled by the hydraulic head gradient, which was generally vertically upward, resulting in a downward flow from the pond to the underlying groundwater. Under these conditions there was minimal influence of groundwater on pond salinity. This, however, could be reversed during periods of pond remediation. The locations of the pond stilling well (BS01) and the underlying groundwater piezometers (BP02 and BP03) are shown in Supplementary Figs. S.5c and 1d. The simulated pond water level data (BS01) confirm that a rise in water level is largely due to rainfall, whereas decreases are caused by evaporation (especially during the dry season), abstraction or seepage into the subsurface. We reproduce the hydraulic heads of BS01, BP02 and BP03 and salt concentrations of BS01, BP02 and BP03 (Fig. 2a,b) in a 2D model. Modelling of the pond water and salt balance provide a reasonable fit to the field data. Discrepancies between the model and the observed data are thought to be due to uncertainties in field measurements, e.g., abstractions by local users. The model parameters (Supplementary Table S.1) were calibrated manually by comparing model outputs to measured hydraulic heads and salinity measurements of BS01, BP02 and BP03 over the simulation period. The hydraulic heads in the underlying groundwater (BP02 and BP03) had a similar trend to the pond water level (BS01) but with fluctuations due to tidal variations in river level (which were not included in the model due to the absence of measured river levels at the site). Sensitivity analysis of the model was carried out for hydraulic conductivity, effective porosity, specific storage, longitudinal dispersity, transverse dispersity, evaporation, river level and pond abstraction by local users. This showed that, whilst changes in hydraulic conductivity and abstraction have a rapid and marked response on pond water level and groundwater hydraulic heads, they have little effect on pond and groundwater salinity. The groundwater hydraulic heads are the most sensitive to changes in the river level and evaporation. Groundwater salinity is sensitive to longitudinal dispersivity, whilst changes in effective porosity and specific storage have minimal influence on pond water level or salinity.

### Remediation scenarios

**Figure 3:**
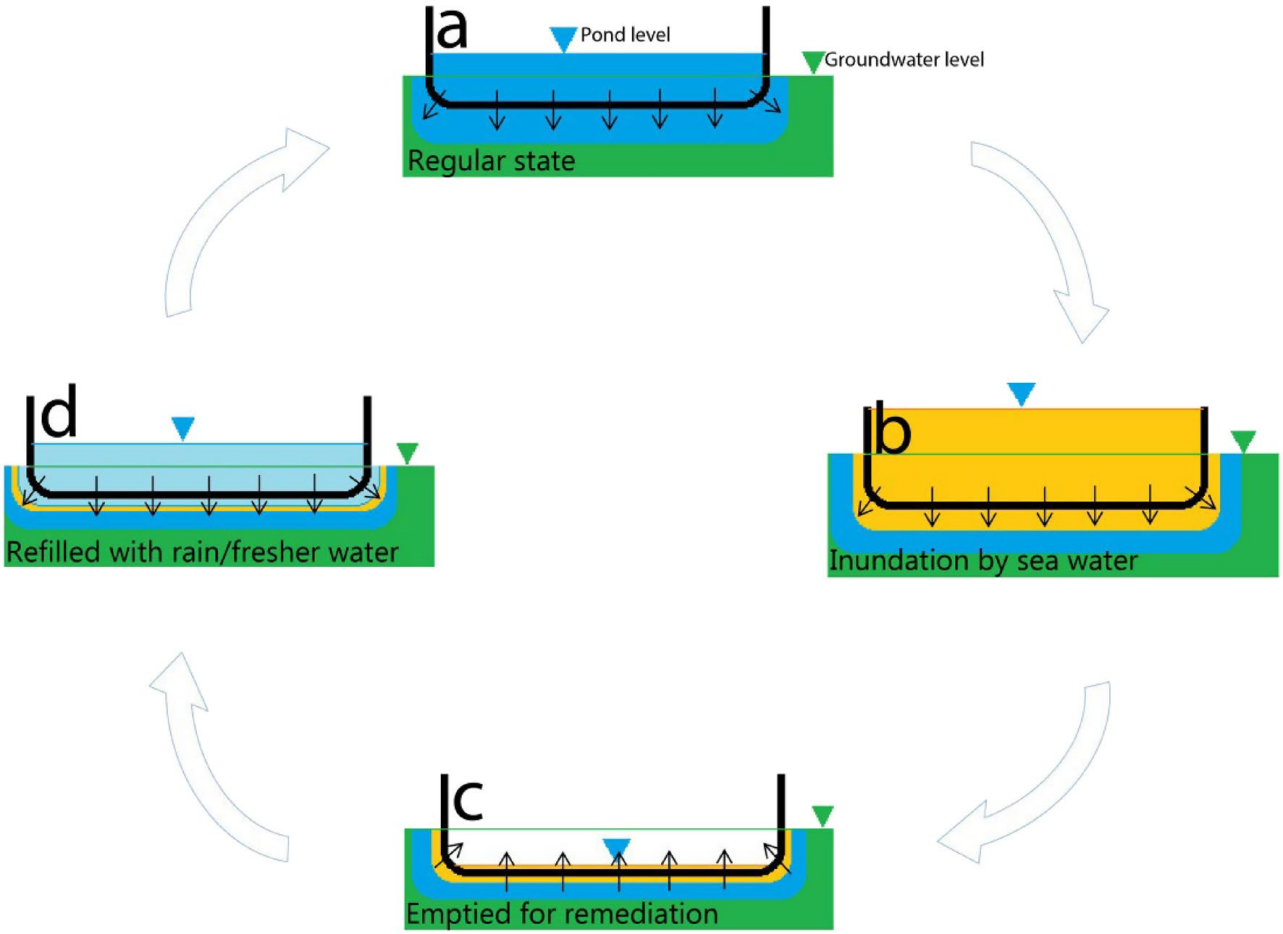
Conceptual mechanism: The salinisation of drinking water ponds in coastal deltas is linked to episodic inundation: (**a**) Normal conditions, water levels in the drinking water ponds are higher than the underlying groundwater, causing a downward flow, leading to freshening of the immediate underlying groundwater. (**b**) Following a storm surge, these ponds are inundated with saline water, which leads to a buildup of salinity in the groundwater immediately below the pond base due to downward infiltration caused by the downward head gradient. (**c**) When saline water is pumped out to remediate the pond, the natural downward gradient changes to an upward gradient for a brief period, bringing back saline water from the underlying groundwater, which mixes with accumulating rainwater in the pond, resulting in significant salinity. (**d**) If, however, the pond is remediated immediately after a storm surge, salinity buildup at the pond base cannot take hold and hydraulic reversal does not become a reason for salinity buildup.

**Figure 4:**
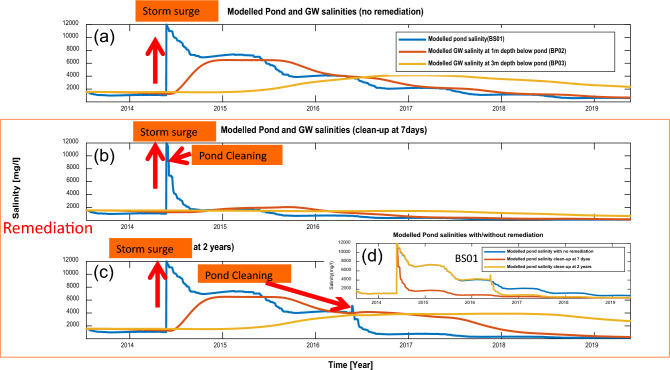
Simulated pond (BS01) and groundwater salinities at depths 1 m and 3 m (BP02 and BP03) below the pond responded to surge events with no remediation (**a**). The pond was cleaned-up at 7 days (**b**) and two years (**c**) after storm events. (**d**) Simulate the response of pond salinities (BS01) to surge events under three scenarios: no remediation, clean-up after 7 days, and clean-up after 2 years.

The model was used to explore three different remediation scenarios: a “no remediation” case and two “pond remediation” cases, one immediate and the other delayed. These aimed to investigate the impacts resulting from a cyclonic storm surge and assess the effect of remediation measures on long-term pond salinity. The concept of pond remediation process is shown in Fig. 3. To avoid climate variation, and due to lack of long-term site data, we undertook five annual cycles using the 2014 rainfall, abstraction, and evaporation data (results shown in Fig. 4 for period 2014–2019). We simulated the effect of the storm surge by perturbing the river water level at B_0_ (Fig. S.1c) (surge levels in Supplementary Fig. S.7). This results in an overtopping of the embankment (next to the pond), flooding the polder interior, including the pond. The long-term effect on pond salinities was investigated through considering different times for clean-up (i.e., remediation). These ranged from no intervention (Fig. 4a), immediate (i.e., 1 week, Fig. 4b) and delayed (i.e., two years, Fig. 4c). Such a long time period prior to pond remediation reflects the experience of many families following a storm surge. For example, from conversations with residents in Dacope Upazila, we learnt that many ponds were not remediated until up to 2 years after the flooding caused by cyclone Aila. This was particularly the case in Polder 32, where the breached embankment was not repaired for many months^27^, resulting in continual flooding with saline water, particularly during high tides. This kept many families away from their homes and meant that their ponds remained unremediated until they were able to return. The timeline for the pond remediation process in this example is illustrated in Figs. 3 and 4c. The initial state is depicted from July 2013 to May 2014 (Figs. 3a and 4c), followed by a storm surge occurring in May 2014 (Fig. 3b). The pond clean-up took place in May 2016 (Fig. 3c), and subsequently, the pond was filled with rainwater after May 2016 (Fig. 3d).

In the long-term (2-year) remediation scenario (Fig. 4c), we simulate a surge inundation with saline river water (salinity 12,000 mg/L) in May 2014. The salinized pond is remediated by emptying after 2 years (May 2016) and allowed to fill with rainwater (salinity 30 mg/L). During the years of high pond salinities, the results show a progressive build up in salinity (due to downward flow from the pond) in the underlying sediments below the pond-base (red and yellow lines in Fig. 4c). Following its remediation through emptying the pond there is a reverse hydraulic gradient which allows the underlying saline groundwater to re-enter the pond (blue line in Fig. 4c and yellow line in Fig. 4d). Although the pond is progressively filled with fresh rainwater during the rainy season (May-Oct), the additional salt from the underlying sediments (over the duration of the reversed hydraulic head gradient, as explained in Supplementary Fig. S.8) maintains a raised level of salinity in the pond (a peak located at May 2016 in Fig. 4c). In contrast, if remediation is undertaken immediately following inundation (in this case, after 7 days in Fig. 4b) salinization is significantly reduced (red line in Fig. 4d) ensuring long-term reductions in pond salinity over many years. This example demonstrates the need for rapid response to pond salinization (pond salinities with no remediation and remediations were compared in Fig. 4d) in order to ensure low salinity pond drinking water in southern Bangladesh, and probably in other deltaic coasts in Asia^22,37^ as well. The need for rapid responses to polder and pond management is likely to become even more critical in future years as climate change results in projected increases in tropical cyclone intensity and frequency^24,25,38^, as explored below.

### Impact of climate change

**Figure 5:**
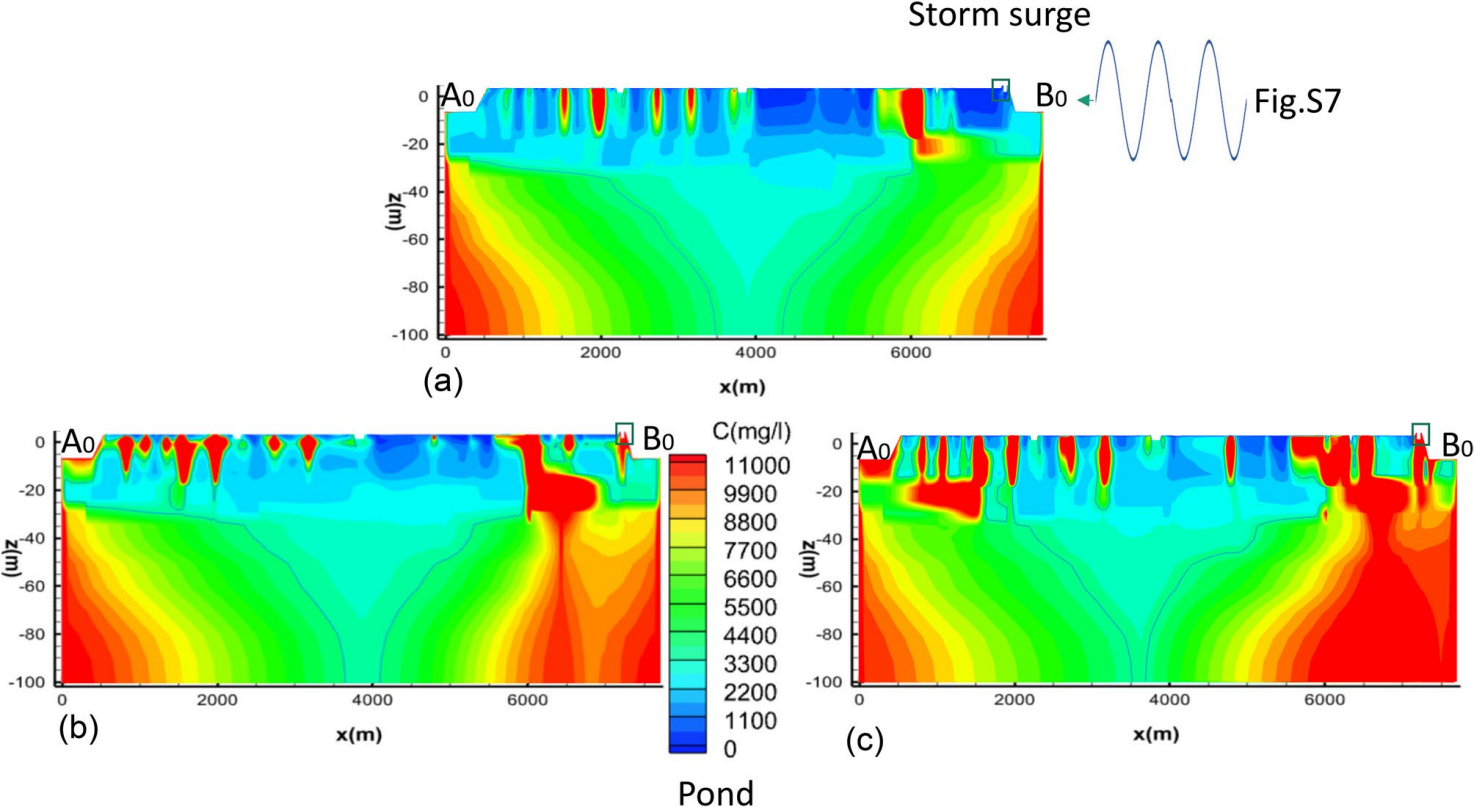
Simulated groundwater salinities to a depth of 100m along the transect shown in Figs. S.1 and S.5, showing impacts after 80 years of repeated storm surge events every 8 years (**a**), 3 years (**b**), every year (**c**). The light blue line represents a salt concentration of 4000 mg/l.

The coastal area of Bangladesh is affected by tropical cyclone induced storm surges approximately every 3–4 years. The events tend to recur at a particular location with a frequency of around 8 to 10 years^39^. To investigate the effects of repeated storm surges on groundwater salinity, we conducted a coupled simulation of the groundwater and surface water system, where an initial non-inundated state is impacted by repeated storm surge occurrences every 8 years for a period of 80 years (Fig. 5a). Even eight years after a storm surge, there are regions of near surface high groundwater salinities located in topographic lows as well as areas with lower salinities due to monsoonal flushing. This is consistent with previous work regarding the impact of storm surges on groundwater salinities in coastal Bangladesh^40^.

To explore the impacts at the polder scale of increased cyclone frequency due to climate change on groundwater salinity a further set of simulations are run for a period of 80 years with storm frequencies of every 3 years, and every year^41^ (Supplementary Fig. S.9). The results show (Fig. 5b,c) that groundwater salinities progressively increase and develop from the surface and infiltrated into the near-surface area over time. In the extreme case (every year), the surge-induced vertical salt infiltration penetrated into lateral saltwater intrusion and pushed the saltwater toe toward inland further (Fig. 5c). These models indicate the effect of climate change on both vertical saline inundation and lateral saltwater intrusion^42^. The results show that episodic storm surges are causing build-up of salinity in shallow groundwater and affecting surface water salinity and drinking water ponds. This is likely to worsen due to projected increased frequency of storm surges^25^ affecting coastal deltas. These cases are all based on one assumption: surge water overtops the embankments. It means that embankments play a crucial role in groundwater and surface water salinities. Therefore, if embankments work adequately to protect polders from tidal and surge water inundations, this could decrease groundwater salinisation considerably.

## Implications

Our results indicate that historic tropical cyclone induced storm surges have contributed to the high groundwater salinities observed in coastal regions of Bangladesh. These saline groundwaters are also enhanced by the presence of saline ponds (e.g., due to shrimp farming). Highly salinity groundwater means that communal ponds are the main alternative low-cost source of drinking water for many living in these coastal regions. These ponds, however, are vulnerable to the impacts from saline water inundation due storm surges. This can result in either the loss of these important local water sources or to prolonged exposure to high salinity drinking water and the consequent health impacts^10,14,15,17^. Simulations show that if these ponds are left to freshen naturally by rainwater dilution it can take around 5 years or more (Fig. 4a). Conversations with pond owners in the three polders in the Dacope Upazila support this result. Residents reported that it typically took between 7 and 10 years for pond salinities to return to levels similar to those prior to a surge event. Rapid clean-up immediately after an inundation event, however, can reduce the salinity problem significantly. As climate change is likely to exacerbate these problems, polder management strategies are therefore vital both by protecting from saline inundation through effective embankment maintenance and repair and by discouraging activities that promote ponding of saline water and irrigation with salty water. Furthermore, when such inundations have occurred, rapid clean-up of drinking water ponds (i.e. within weeks) can greatly reduce impacts from exposure to high salinity drinking water. Whilst this work has focussed on Bangladesh, the results are applicable to other Asian deltas, such as the Mekong and Irrawaddy^22^, where similar drinking water sources are vulnerable to impacts from tropical cyclone storm surges.

## Methods

### Salinity monitoring

Purpose built drinking water ponds are numerous in southwest Bangladesh and many have excess levels of salinity^43^. We surveyed a number of ponds in Polders 31, 32 and 33 in Dacope Upazila, Khulna district, southwest Bangladesh (Figs. 1, S.1 and S.3). The ponds are typically constructed with a steep clay embankment, which generally prevents rainwater run-on, apart from some sites where small breaks provide access for domestic animals. We monitored a relatively large (40 m × 100 m) pond, DAB^9^ in Polder 31 over an 18-month period (07/2013–01/2015). The pond is located adjacent to a tidal river. On the north side is a shrimp farm, while to the west is a settlement. The pond is used by local people for drinking and other household purposes but not for bathing. Pond water is also used in an aquifer storage and recovery (ASR) scheme operated in the settlement area. Direct rainfall is the main source of inflow to the pond. Exchange of water between the pond and underlying silty sediments can occur due to head differences between the pond and the underlying groundwater. Episodic inputs of saline water to the pond result from storm surge inundations. Outflows from the pond are due to evaporation and from abstractions either for drinking purposes or ASR input. Major abstractions of saline water can also take place following saline inundation, as part of a clean-up programme.

A platform was constructed in the centre of the pond (Fig. 1c). The platform also provided access to an adjacent stilling well for measuring pond water level and two piezometers, screened at 1 and 3 m below the pond base, measuring groundwater levels in the sediments underlying the pond (Fig. 1d). Piezometer transects were also installed to measure the interaction of the pond with the river and adjacent groundwater.

Vertical exchange between pond and groundwater was determined using a stilling well and two piezometers, screened at 1 and 3 m below the pond base. These were all located on a platform in the centre of the pond (Fig. 1c,d). The platform also supported a tipping bucket rain gauge and a purpose-built evaporation pan. The system was fitted with automatic loggers (Solinst) that measured rainfall, pond, and groundwater level (1 m), and fluid electrical conductivity at 15-min intervals. Piezometers monitoring groundwater levels and fluid electrical conductivity external to the pond area, along with a flow meter for ASR abstractions, were daily manually recorded. Piezometer and stilling well water levels are all referenced to a local datum. Salinities were calculated from electrical conductivity using the following equation: salt (mg/l) = 0.48EC (µS/cm), which was derived from laboratory analyses. In addition to site measurements, evaporation data for the duration of the study were also provided by the Bangladesh Meteorological Department for Mongla, located 10 km from the pond.

A bathymetric survey of the pond was conducted and empirical relationships of water level to wetted area and volume of water determined. Geological characteristics of the underlying sediments were carried out by grain-size analysis of the drill cuttings and auger samples. The grain size analyses were augmented with measurements of bulk densities to facilitate estimation of hydraulic conductivities^9^.

### Numerical modeling

#### Model development

Although the field monitoring provides information on interaction of pond salinities on groundwater, to understand the wider, long-term impacts of tropical cyclone induced storm surges on pond and groundwater salinities requires numerical modelling^35^. To achieve this, a 2D cross-section model (A_0_B_0_ Fig. S.4) of the drinking water pond and surrounding polder at the DAB site (Fig. S1) was developed using HydroGeoSphere^35,44,45^. HydroGeoSphere (HGS) is a fully coupled surface and variably saturated subsurface flow and transport model. Subsurface flow is represented by Richards’ equation^46^, with pressure potential as the independent variable. The hydraulic properties of the soil and underlying sediments are represented using the non-hysteretic, parametric relationships of van Genuchten^47^, for the soil water characteristic, and Mualem^48^, for the unsaturated hydraulic conductivity. Density-dependent transport of total salt concentration (i.e., salinity) in the subsurface is modelled using the advection–dispersion equation^44,49^, where the hydrodynamic dispersion is the sum of apparent molecular diffusion and mechanical dispersion. The latter represented using “Fickian” longitudinal and transverse dispersivities (see Supplementary Eq. S.1 for further information). Overland flow is represented by the diffusion wave approximation of the Saint Venant equations. The surface and subsurface components are coupled by the addition of a thin layer (here set to 10^−4^ m^50^). This approach allows exchange of water (and salt) between the surface and surface domains to be calculated based on a “Darcy” representation of flow, driven by the head difference between the surface water level and the hydraulic head in the upper most soil layer. The model parameterisation was a combination of direct measurement and calibration conditioned on the field data. Further details are provided in the Supplementary Information.

#### Model application

Surface land use and topography was determined from surveyed data in the vicinity of the pond and from information available from Google Earth^51^. Near surface geological data were provided from logs obtained from the near surface boreholes drilled around the pond and supplemented by previous work^8,9,39,52^.

The surface of the model included the drinking water pond, the polder embankment, drainage canals, and adjacent river (see in Supplementary Fig. S.1a) and was set as 7650 m (wide). The base of the model was set at a depth of 100 m. This was considered sufficiently deep to support the use of a no-flux boundary^39^. The surface domain was discretized using 512 rectangular 1D finite elements. The surface nodes coincide with top nodes of the subsurface domain (Fig. S.5). The subsurface domain was discretized into 33 variable thickness layers, giving 16,896 rectangular elements, with a finer resolution near the surface to ensure the exchange of water and salt between the surface and subsurface (which is a key process) was appropriately represented (Figs. S.4 and S.5).

The height of the polder is 5 m above sea level (masl), while the elevations of the surrounding agricultural land, pond base and riverbed are 4masl, 2.3masl and -6masl, respectively (Table S.2). The river level data for the period 2013–2015 were obtained from measurements at Mongla. Further information about land use heights, canal, and river depths, and lithological data for the study area can be found in Supplementary Tables S.1 and S.2 and Figs. S.4 and S.5.

According to the lithological analysis^39^, there is a 5–15 m thick near-surface silty clay layer, across the study area. Below, around 25–35 m deep, is a 5–10 m thick discontinuous silty clay layer. Between these layers is a layer of fine to medium sand, which functions as a near-surface aquifer. Numerous silty clay layers intercalated with finer sands form a deeper aquifer (35–100 m deep). These lithological data are used to characterize the hydrogeological regime as shown in Supplementary Fig. S.4.

To implement the model, hydraulic parameter values were derived from measured data and literature values, supplemented through model calibration. These are summarized in Table S1. Effective porosity and specific storage were suggested from a previous study^39^. Residual saturation, inverse of air entry pressure and pore-size distribution index were estimated by the computer program Rosetta^53^. Hydraulic conductivity for the silty clay and sand layers were calibrated by comparing measured and simulated pond water and groundwater levels.

The boundary conditions for surface and subsurface domains are shown in Fig. S.4 and Table S.3. Net infiltration is assumed to be 200 mm/yr^39^ (about 10% of annual rainfall) which was applied across the surface domain. The base of the model is a no-flow boundary. The water depth of the riverside boundary^50^ in the surface domain is given as:

$${\text{d}} = {\text{SRL}}\left( t \right){-}{\text{z,}}$$

where SRL represents saltwater river level, which is set at 3masl,

whilst, the value in subsurface domain was given as:

$${\text{h}}^{{\text{f}}} = \left[ {{\text{SRL}}\left( {\text{t}} \right) - {\text{z}}} \right] + {\text{ z}},$$

The model was initialized by running to steady state. Following initialization, it was driven using daily time series data of evaporation, rainfall, and pond water abstraction for 2014 during non-surge conditions. To simulate the effect of a storm surge, the river levels (B_0_ in Fig. S.4) were replaced by surge levels. These surge water levels (see Supplementary Fig. S.7) were derived from the University of Hawaii sea level center^54^. The 2014 evaporation and rainfall data were repeated to enable multi-year simulations. Climate change, resulting in warmer seas, is likely to result in an increase in the occurrence of severe tropical cyclones in the Bay of Bengal^25,34^.

### Supplementary Information

Supplementary Information.

## Data Availability

Field data used during the current study are available from the corresponding author on reasonable request. Input files for the HGS simulations are also available from the corresponding author on reasonable request.
