# Supplementary material for: Salinisation of drinking water ponds and groundwater in coastal Bangladesh linked to tropical cyclones
Source: Sci Rep. 2024 Mar 3;14:5211. doi: 10.1038/s41598-024-54446-6 (PMC10909877; doi:10.1038/s41598-024-54446-6)
Supplement: Supplementary file 1 — Supplementary Information. [file 41598_2024_54446_MOESM1_ESM.docx]

Supplementary information

**Salinisation of drinking water pond in coastal Bangladesh is linked to tropical cyclones**

Chi-San Tsai,^1,2*^ Mohammad. A. Hoque^1, 3^, Paolo Vineis^4^, Kazi Matin Ahmed^5^, Adrian P. Butler^1^

^1^Department of Civil and Environmental Engineering, Imperial College London, UK

^2^Department of Environmental Systems Graduate School of Frontier Sciences, the university of Tokyo, Japan

^3^School of the Environment, Geography & Geosciences (SEGG), University of Portsmouth, Portsmouth PO1 3QL, UK

^4^MRC-HPA Centre for Environment and Health, Department of Epidemiology and Biostatistics, Faculty of Medicine, Imperial College London, UK

^5^Department of Geology, University of Dhaka, Bangladesh

*Email: a.butler@imperial.ac.uk; Tel: +44 (0) 20 7594 6122

**1. FIELDWORK MONITORING**

The study area is in the Dacope Upazila region of the southwest coastal district of Khulna (Figure S.1(A) and (B)). Rainfall, pond water levels, groundwater levels, evaporation, and salt concentrations are shown in Figure S.2. The seepage rate was calculated by a water balance from site measurement as shown in Table S.1. Electrical conductivity for water salinity was measured in (µS/cm) for 15 minutes intervals during July 2013-Jan 2015 (Figure S.2). After Cyclone Aila, measurements were taken for several ponds in Polders 31, 32, and 33 (Figure S.3).


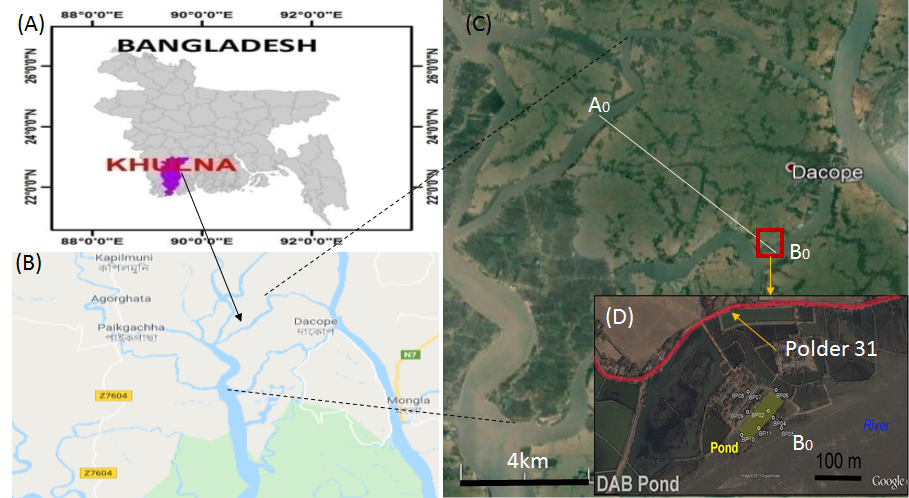


**Figure S.1: (A), (B) and (C): The study area is in the Dacope Upazila region of the southwest coastal district of Khulna in southwest coastal regions of Bangladesh (D) The study area is located outside Polder 31. (Images sourced from Google Earth Pro, accessed 10 April 2019)**

**Table S.1 A water balance calculation for the DAB pond 15-July 2013-14 July 2014.**

| Annual rainfall | 2201 [mm] |
| --- | --- |
| Annual evaporation | 1234 [mm] |
| Annual abstraction | 339 [mm] |
| Annual seepage | 628 [mm] |


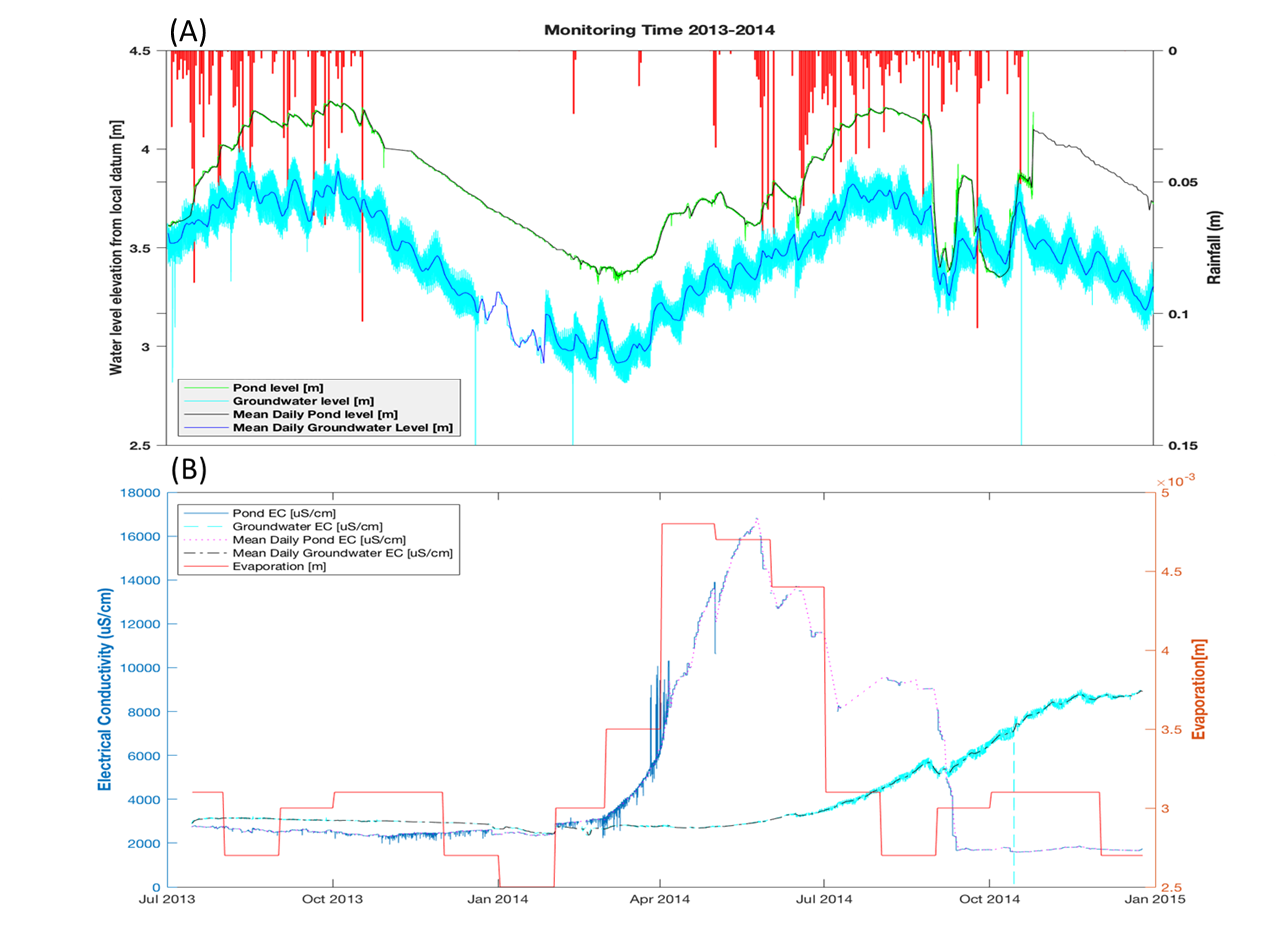


**Figure S.2 (A): Pond water level (referenced to a local datum), groundwater levels, mean daily pond water levels, mean daily groundwater levels and rainfall data from 14- July- 2013 to 24 December-2014. (B) The pond and groundwater electrical conductivities (EC), mean daily pond and groundwater electrical conductivities (EC), and evaporation data over the same period.**


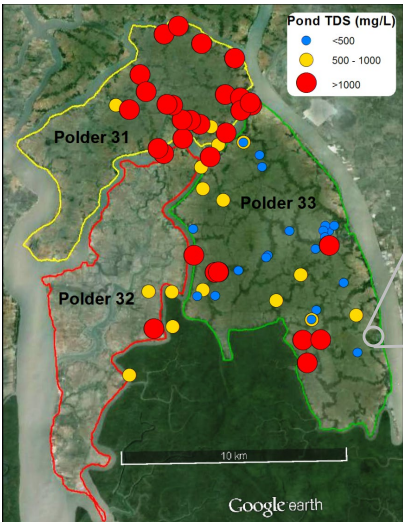


**Figure S.3 Red circle represents pond salinity higher than 1000mg/l, yellow circle pond salinity is between 500mg/l to 1000mg/l and blue line represents pond salinity lower than 500mg/l. The ponds in Polder 33 had lower salinity than the ponds in Polder 31 and 32 during Cyclone Aila, as Polder 33 was less affected by the storm surge. (Image sourced from Google Maps (2020) Bangladesh, available at: http://maps.google.co.uk, accessed: 5 July 2020)**

**2. NUMERICAL MODELLING**

Hydrogeosphere (HGS) couples surface and variably saturated subsurface flow and solute transport equation^36,37^. These equations can be found in HGS manual^37^. HGS uses the dual node approach (similar to Darcy’s law given by head differences) to fully couple surface and subsurface domains by a thin layer. The thickness of this thin layer is called the coupling length which is 10^-4^m^36,42^. This value was decided by sensitivity analysis. The analysis was carried out by using modified Henry problem^42^. HGS used this approach to calculate water exchange flux Q_ex_ (L/T) between two domains.

$Q_{ex}=\frac{K_{r}K_{zz}}{l_{ex}}\left( h-h_{0} \right)$ S.1

where Q_ex_ is the exchange flux between the surface and the subsurface (L/T), k_r_ is the relative permeability of the surface (-), *K_zz_* is the saturated hydraulic conductivity of the underlying porous media (L/T),$l_{ex}$ is the coupling length (L), and h and h_0_ are the hydraulic heads of the subsurface and surface. They are equal to the pressure head plus the elevation head of surface and subsurface domains. If h>h_0_, it means water flows from the surface to the subsurface domain while h<h_0_, it means water flows from the subsurface to the surface, respectively.

Dual-node approach (Eq.S 1) is used to define the solute exchange terms Ωex between surface and subsurface domains. The exchange term can be expressed as:

$\Omega_{ex}=C_{ups}Q_{ex}$ S.2

Where$C_{ups}$ is upstream concentration of the exchange flux

$C_{ups}\mathbf{=}C if Q_{ex}>0$

${=C}_{inflow}if Q_{ex}<0$ S.3

If exchange flux >0 it means groundwater discharge from the aquifer to the river and the concentration of the exchange flux equals to the concentration of the water in the aquifer ($C_{ups}\mathbf{=}C)$. If exchange flux < 0 it means that river water flows into the aquifer and the concentration of the exchange flux equals to concentration of the salty river ${(C=C}_{inflow}$).

Salt exchange between the surface and subsurface domains is determined only by advection for the dual-node approach of representing these two domains. Advection term occurs with the fluid flows between the two domains and Q_ex_ is calculated in the flow equation. For the case if the hydraulic heads in subsurface domain are equal to that in the surface domain, then, according to Eq.S.1, the term Q_ex_ becomes equal to 0 and then the salt exchange is given by term Ω_ex_ becomes to a diffusion-type of salt exchange between the two domains^42^.

This numerical approach was used to solve the model based on the fieldwork measurements.

**2.1 Lithological analysis**^12,42,43^

As the monitoring system is in the pond, a 2D cross section was conducted along A_0_B_0_. According to the field based data^6,44,45^ (Figure S.6(a)) which is only about 3 km far from the A_0_B_0_, there is a silty clay layer about 5-15m thick at the near-surface shallow part of the study area. Below the superficial layer, there is a dis-continuous silty clay layer. The discontinuous layer, located at a depth of 25m to 35m, has a thickness ranging from 5m to 10m. There is a sandy layer including very fine sand to medium sand between these layers. The discontinuous silty-clay layer separates the shallow aquifer from the deeper aquifer. Numerous silty clay layers are intercalated with finer sands at deeper aquifer. These lithological data are used to characterize the hydrogeological regime. Therefore, the 2D cross-sectional model was conducted based on these data.

**2.2 2D cross–sectional model development**

The 2D cross-section along A_0_B_0_ was developed across the drinking pond and the polder in DAB site (Figure S.1(C)) and was set as 7650m (wide) and a datum at 100m deep (Figure S.4). The surface domain is represented by 1D rectangular finite elements. The surface nodes coincide with the top nodes of subsurface domains.


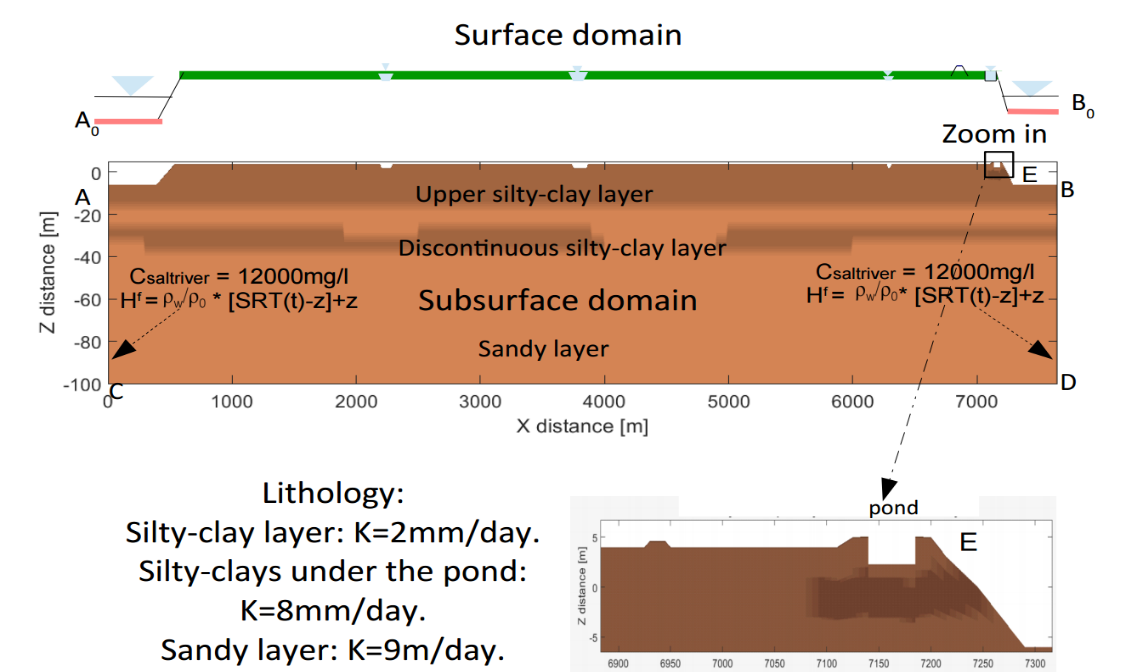


**Figure S.4 HydroGeoSphere surface and subsurface domain coupling. Boundary conditions for surface and subsurface domains. The LHS boundary (BD) and the RHS boundary (AC) are riverside boundaries.**

The three observed points (labeled BS01, BP02 and BP03, one is at the drinking pond, the others are at 1 and 3 meter depths below the drinking pond (Figure S.5(c)) were set, which correspond to the measurement data. The land use features, and elevation are based on Google Earth and field data. Figure S.5 also shows the elevation and land use features of the surface domain of a cross section A_0_-B_0_. To emphasize the surface features and the measured points, Figure S.5 is exaggerated vertically and shrunk horizontally. The height of the polder is 5 m.a.s.l (meters above sea level), while the elevation of agricultural land, drinking pond bed and riverbed are 4m.a.s.l, 2.3 m.a.s.l and -6m.a.s.l. The elevation of the bottom layer is assumed to be -100m.a.s.l. The river level (3m) is chosen from the measured data at a station in Mongla during 2013-2015 which is about 5km from the drinking pond. More specific information about height of land use, depth of canal and river and lithological data below this study area are shown in Table S.1 and Table S.2.

**2.3 Model parameters**

The simulated parameters were derived from measured data and the model calibration and were supplemented by literature values. These simulation parameters are summarized in table S.1 and specific storage was obtained from the literature^41^. Residual saturation, the inverse of air entry pressure and the pore-size distribution index were in accordance with the literature^36^ and can be found in the table that is generated by a computer program named Rosetta. Initial hydraulic conductivity for silty clay was also estimated by Rosetta^46^. Therefore, the program used referenced soil texture data and bulk density^6^ to estimate a proper range of hydraulic conductivity. Then, the range of hydraulic conductivity was tested by comparing the measured pond water level and groundwater levels with simulated levels until the calibrated value was obtained, as measured data is close to simulated data (the root mean square error (RMSE) is lower than 0.1). Hydraulic conductivity for sand was referenced from supplementary data in previous studies^45^.The simulated parameters are summarized in Table S.1.


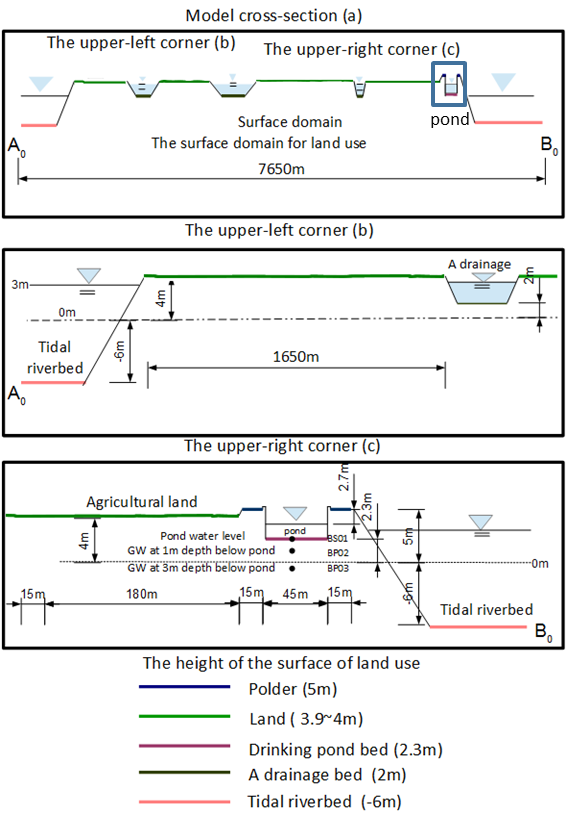


**Figure S.5: (a) Elevation and land use features of the surface domain of the cross section A_0_-B_0_ in Figure S.1(C). (b) Upper-left corner of A_0_-B_0_ cross section. (c) Upper-right corner of A_0_-B_0_ cross section.**

**Table S.1 Summary of simulated parameters for DAB site.**

|  | symbol | | value | Reference |
| --- | --- | --- | --- | --- |
| **Parameters from literature values** |  |  | |  |
| Effective porosity | *θ* | 0.3 | | 41 |
| Specific storage | *S_s_* | 1x10^-4^ | | 41 |
| Molecular diffusion coefficient | *D_0_* | 9x10^-4^ m^2^/day | | 36 |
| Reference fluid viscosity | ν | 1.124x10^-3^kg/m*s | |  |
| Seawater density | *ρ*_s_ | 1026 kg/m^3^ | |  |
| Freshwater density | *ρ_0_* | 1000 kg/m^3^ | |  |
| Freshwater concentration | C_0_ | 0 mg/l | |  |
| Seawater concentration | C_s_ | 35000 mg/l | |  |
| Residual saturation | *S_wr_* | 0.024/0.079 | | 46 |
| Inverse of air entry pressure | α | 0.7/1.3m^-1^ | | 46 |
| Pore-size distribution index | β | 1.58/1.50 | | 46 |
| **Parameters from calibration** |  |  | |  |
| Hydraulic conductivity(silty-clay) | K_1_ | 2x10^-3^m/day | | From the measured |
|  | K_2_ | 8x10^-3^m/day | | Pond &GW level |
| Hydraulic conductivity(sandy) | K_3_ | 9 m/day | | 6 |
| Longitudinal dispersivity | D_L_ | 0.2m (silty-clay) | |  |
|  | D_L_ | 0.8m (sand) | |  |
| Transverse dispersivity | D_t_ | 0.05m | |  |
| **Parameters from literature values at DAB site** | | | |  |
| River concentration | C_sr_ | 12000mg/l | | 45 |
| Salty river density | ρ_sr_ | 1008.342kg*m^-3^ | |  |

Longitudinal and transverse dispersivity are usually suggested by the reference value^43^. However, in this case, these two parameters were calibrated by comparing the measured pond and groundwater salinities with the simulated salinities. River water salinity was obtained from a previous investigation^45^ and based on the average seasonal values of Bangladesh Water Development Board (BWDB) data at the Chalna station. The elevation and width of land-use features were provided by Google Earth. Therefore, a 7,650m length of the cross-section was chosen for this 2D model. The height of the polder was 5 m.a.s.l, while the elevation of agricultural land, drinking pond bed and riverbed were 4 m.a.s.l, 2.3 m.a.s.l and -6 m.a.s.l, respectively. The elevation of the bottom layer is assumed to be 100 m.a.s.l. The mean river level (3m) is chosen from the measured data at a station in Mongla during 2013 to 2015. Table S.2 represents the elevation of the surface and land use^33^ based on Google Earth^43^. Discontinuous layers in Table S.2 were conceptualized from previous work^6,44,45^. More specific information about the height of land-use, depth of the canal and river and lithological data about the following study is shown in Tables S.1 and S.2.

The subsurface elements are represented by different lithology and include silty clay, sand and discontinuous silty clay layers. More specific information about these layers is shown in Table S.2. The hydraulic conductivity of these layers was assumed to be 2mm/day and 8mm/day for the silty clay after calibration and 9m/day for sandy layers, as shown in Figure S.3. The hydraulic conductivity of silty layers was obtained during the model calibration. Before introducing the inundation event, the result of the model initialization was regarded as a basis for the inundation event model.

**2.4 Grid resolution**

The single layer of horizontal variable length and unit width elements is used for this 2D cross-section. Rectangular elements were used for the 2D surface domain, which is basically a 1D representation of the surface. The surface domain is discretized by 512 rectangular elements. For the vertical grid resolution, from the top layer to z=-0.7m, it discretised by 15 layers, for example, from the pond base (z=2.3m) to z=-0.7m, dz=20cm. Then, from z=-0.7m to z=-6.9m was discretised by 5 layers and the thickness of the layer is two times of the thickness of its upper layer (i.e. dz=0.2, 0.4, 0.8, 1.6 and 3.2m). From z=-6.9m down to the bottom (z=-100m) was discretised by 8 layers.

**Table S.2 Elevation/depth and width of land-use and features (surface) and lithology (subsurface).**

| Domain | Types | Width | Elevation/depth |
| --- | --- | --- | --- |
| Surface | Tidal river | 390m | 3m above MSL/8m |
| Surface | Polder | 15m | 5m above MSL |
| Surface | Canal | 75m/105m/15m | 2m above MSL/2m |
| Surface | Agricultural land | 6630m | 4m above MSL |
| Surface | Pond | 45m | 2.3m above MSL |
| Subsurface | Upper silty-clay | 7650m | Top to -15m |
| Subsurface | Sandy layer | 7650m | -100m to -15m |
| Subsurface | Discontinuous silty-clay | | -25m to -35m |
| Subsurface | silty-clay layer | 1900m | -27m to-25m |
| Subsurface | silty-clay layer | 1500m | -35m to -31m |
| Subsurface | silty-clay layer | 750m | -34m to -27m |
| Subsurface | silty-clay layer | 1400m | -29m to -23m |
| Subsurface | silty-clay layer | 2200m | -33m to -28m |
| Subsurface | silty-clay layer | 1400m | -28m to -23m |
| Subsurface | silty-clay layer | 1500m | -30m to -24m |
| Subsurface | silty-clay layer | 1100m | -34m to -28m |
| Subsurface | silty-clay layer | 750m | -28m to-24m |
| Subsurface | silty-clay layer | 200m | -3m to 1m |

**2.5 Boundary conditions and initial conditions**

All boundary conditions for surface and subsurface domains are shown in Figure S.5 and Table S.3.

**Table S.3 Boundary conditions for surface and subsurface domains.**

| Domain | Domain | Head/Flux | Transport |
| --- | --- | --- | --- |
| A_0_ | Surface | d= SRL(t)-z_A0_ | C=C*_saltyriver_* |
| B_0_ | Surface | d= SRL(t)-z_B0_ | C=C*_saltyriver_* |
| A_0_B_0_ | Surface | q_p_= 200mm/yr | - |
| Riverside(BC) | Subsurface | h^f^=[SRL(t)-z]$\frac{\rho_{s}}{\rho_{0}}$ + z | C=C*_saltyriver_* |
| Riverside(AD) | Subsurface | h^f^=[SRL(t)-z]$\frac{\rho_{s}}{\rho_{0}}$ + z | C=C*_saltyriver_* |
| Bottom | Subsurface | Impermeable | $\frac{\partial c}{\partial n}$ =0 |

SRL(t) is salty river levels

**2.6 Model initialization**

To initialize groundwater salinity distribution for further investigation, the model was run for 100 years. It seemed a reasonable period as the polder has been in place since the 1960s and the age of the lower shallow aquifer (70m to 100m deep) is about 100 years^30,31^. Note that the sea level rise and other elements of change in climate were not considered since this was used to form the basis for further simulations.


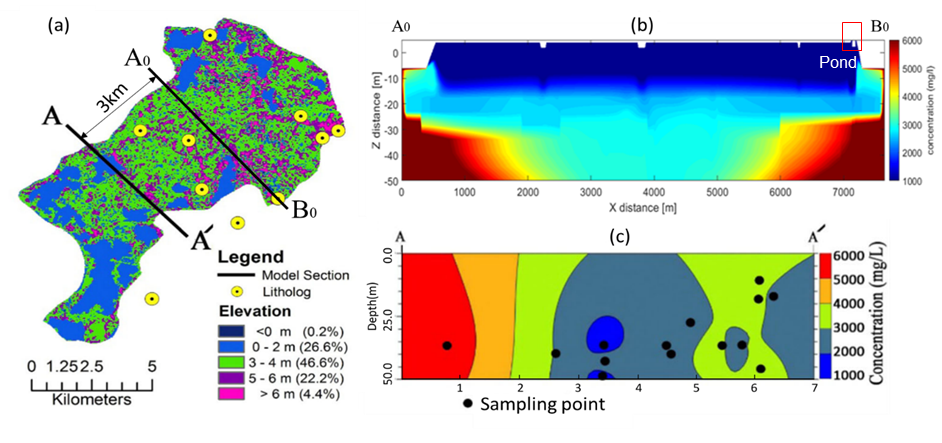


**Figure S.6 (a) Elevation map cited from previous investigation^33^. (b) Uncalibrated groundwater salinities along section A0 – B0. (c) Measured groundwater salinities along A – A’^33^. Groundwater salinities were generated using linear kriging interpolation.**

**2.7 Comparison of measured groundwater salinity along A-A’ and simulated groundwater salinity along A_0_-B_0_**

The measured groundwater salinities^44^ are along A-A’ which is only about 3km from the drinking pond as shown in Figure S.6(c). The hydrological and hydrogeological properties of cross-section A-A’ were assumed to be similar to cross-section A_0_-B_0_. Some basic parameters of the cross section A_0_-B_0_ are referenced from previous study^33^. The initial condition of the groundwater salinity was 3000mg/l based on measured groundwater salinity^33^ which is variation between 1000mg/l-6000mg/l. The range of salt concentration is from 0mg/l to 6000mg/l. The model is considered as a preliminary model for calibrating model parameters. The range of salt concentration is from 1,000mg/l to 6,000mg/l. A comparison of the measured groundwater salinities along cross-section A-A’^45^ showed that the RHS of the simulated groundwater salinity distribution was different from the measured one. This is because the salinity of the salty river in the field varies, whereas tidal river salinity in the model is a constant value. The simulated result is thought to be acceptable, particularly as the model had not been calibrated.

**2.8 Conceptual model for surge events**

The 2014 rainfall, abstraction and evaporation data were repeatedly used for 5 years (regarded as 2014-2019) and we introduced cyclonic storm surge and pond clean-up (i.e., remediation) related pumping in two different times (i.e., 7 days and 2 years). SRL in the Eq S.4 and S.5 represents the surge levels. The effects of the cyclone lasted roughly 18 hours and its tidal surges ranged from 2.3 m to 6.8 m, as shown in Figure S.7. These surge water levels were introduced to the river level at B_0_.


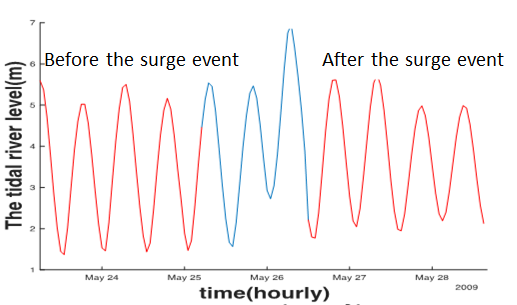


**Figure S.7 Storm surge levels at Chittagong station. The red line represents before/after the surge event, and the blue line represents surge levels.^45^**

**2.9 Reversed head gradient between the pond and groundwater after clean-up the pond**

The results of scenario 2 (Figure 4(c)), which involved cleaning up the pond two years after the surge event, demonstrate a reversal in the head gradient beneath the pond after the remediation process (Figure S.8(a)). This reversal occurred through three-month periods, leading to an influx of saline groundwater over the next three months. Despite the influx of salt to the pond, this is countered by enhanced dilution from monsoonal rainfall (Figure S.8(b)). As a result, the pond water level rises, and the upward flow is reduced. This result shows the importance of monsoon rains in relation to the impact of storm surges and the timing of remedial responses.

**Figure S.8(a) Hydraulic heads of pond and groundwater at a depth of 1 m below the pond base and (b) pond salinity response to the remediation and monsoonal rainfall (May, June, and July) as the pond was clean-up at 2 years after the surge event. The blue bars show daily rainfall (in m). Note that the timeline presented does not reflect real-time events. It is assumed that the storm surge event occurred in May 2014.**

**2.10 Effect varying frequency of recurrent cyclones**

The study area is periodically affected by tropical cyclones as mentioned in the main text. We developed three scenarios of different frequencies of recurrent cyclones (Figure S.9) that hit the study area. Although climate change was discussed in this study, it was considered the cyclone frequency only, regardless of other climatic factors such as rainfall and evaporation. The results have been discussed in simulation for the effect of climate change on the groundwater salinity distribution.


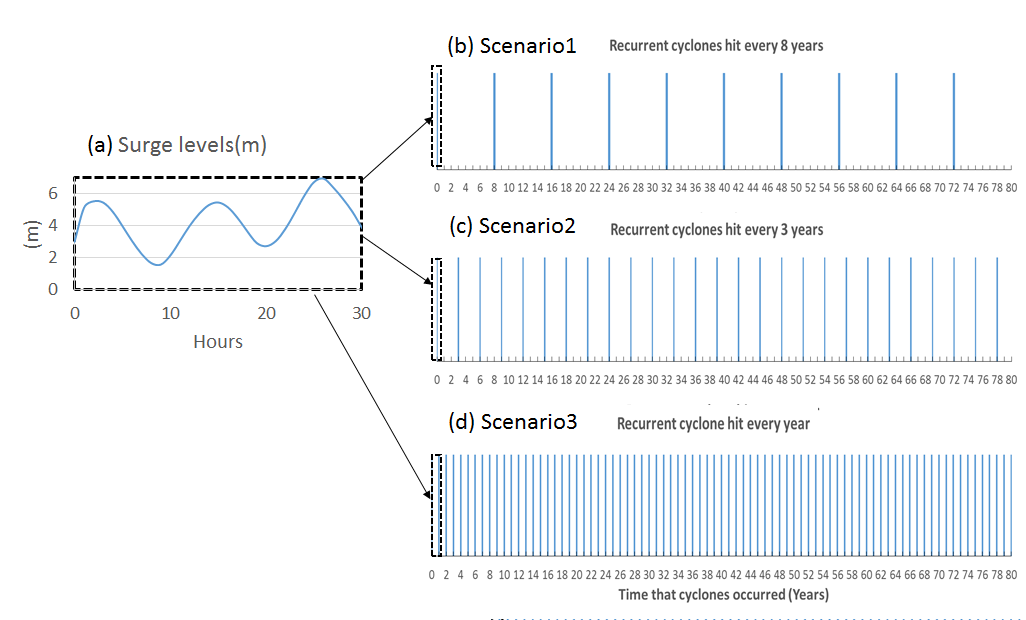


**Figure S.9 (a) Storm surge levels change with time during the storm^34^. (b) Scenario 1: Recurrent cyclones hit the land every eight years. (c) Scenario 2: Recurrent cyclones hit the land every three years. (d) Scenario 3: Recurrent cyclones hit the land every year. Note: time scale of (a) is hours, whereas for (b), (c) and (d) it is in years.**
